# Hear No Virus, See No Virus, Speak No Virus: Swiss Hotels’ Online Communication Regarding Coronavirus

**DOI:** 10.1007/978-3-030-65785-7_43

**Published:** 2020-11-28

**Authors:** Laura Zizka, Meng-Mei Chen, Effie Zhang, Amandine Favre

**Affiliations:** 1 Department for Informatics, Technical University of Munich, Garching bei München, Bayern Germany; 2 Smart Tourism Education Platform (STEP) College of Hotel and Tourism Management, Kyung Hee University, Seoul, Korea (Republic of); 3 Department of Tourism and Service Management, MODUL University Vienna, Vienna, Wien Austria; Ecole Hoteliere de Lausanne, HES-SO University of Applied Sciences and Arts Western Switzerland, Lausanne, Switzerland

**Keywords:** Swiss hotels, Coronavirus, De-confinement, Facebook, Situational Crisis Communication Theory (SCCT)

## Abstract

Tourism is a lucrative business, and Swiss hotels rely heavily on international clientele to book their rooms. The Coronavirus pandemic has halted travel and hotel stays from March to June 2020. Based on Situational Crisis Communication Theory (SCCT), this paper investigates the messages Swiss hotels have posted on their official websites and Facebook pages to reassure guests that it is safe to book rooms in Switzerland again. The findings from 73 independent 4 and 5-star hotels show that most hotels did not publish messages regarding the Coronavirus or the measures they have taken; instead, the hotels posted positive messages about reopening their rooms and services. Official hotel websites emphasized deals and offers while the Facebook pages concentrated on enthusiastic ‘welcome back’ messages. The findings presented here contribute to the literature by offering the first results of a larger project on communication during the de-confinement stage of a pandemic.

## Introduction

Tourism in Switzerland is a lucrative business, providing many jobs to the local communities and contributing significantly to the local economy. According to the official statistics published in 2019, there are 28 985 hotel and restaurant establishments with 175 489 full-time employees and 7845 trainees who generate CHF 44.7 billion in total revenue, CHF 16.6 billion from foreign tourists in Switzerland through hotels, restaurants, and transportation. The tourism industry is one of the largest export industries in Switzerland, with 4.4% of export revenue [1]. Thus, the tourism industry in Switzerland relies heavily on foreign travelers. In 2019, the Swiss hotel industry registered 39.6 million overnight stays across the country [2]. It was a profitable year for Swiss tourism, and the expectations for 2020 were positive until the first reports of the Coronavirus in China.

As word of the Coronavirus spread, the Swiss tourism industry began to make predictions regarding the 2020 tourism season. In a message published in January 2020, one tourism expert stated: “In the coming weeks, Switzerland Tourism expects a 30–50% reduction in the number of Chinese visitors to Switzerland” [3]. Based on 2018 figures, this could represent 70,000–100,000 overnight stays per month. As Chinese visitors are among the biggest spenders when holidaying in Switzerland, paying out on average CHF380 per person per day, this pandemic could be a dire situation for Swiss tourism affecting popular Swiss destinations such as Geneva, Zermatt, Interlaken, Lucerne, and Zurich. As hotels are heavily affected by international travel, they are likely to feel greater repercussions from the Coronavirus restrictions than restaurants [4].

By March 2020, a survey conducted by Valais University of Applied Sciences and Arts predicted that the tourism industry revenue could suffer an 18% loss due to the restrictions and, in some cases, closure of hotels and restaurants. While the study predicted up to 6 billion CHF losses for the tourism industry in 2020, the hotel sector alone would lose as much as 2 billion CHF between March and May (the time this study was conducted) [4]. In the same survey, bookings had dropped by 69% for March, 90% for April, and 73% for May. The President of the Swiss Hotel Association, Andreas Zullig, estimated that 5% of Swiss hotels (from 200–250) would not survive the pandemic [4].

This paper examines the messages during and after the confinement of 73 Swiss 4 and 5-star independent hotels to analyze how they communicated with their customers regarding the Coronavirus and whether they applied appropriate crisis communication strategies. The purpose is to gauge whether the hotel messages were effective in reassuring the customers that Swiss hotels are prepared and ‘safe’ to book during their holidays. Their messages posted on their official websites and their Facebook pages are measured against Situational Crisis Communication Theory (SCCT). A content analysis of their messages was conducted to create word combinations and visuals through Wordji. This paper concludes with the implications of using social media and company websites to communicate with customers in the time of a crisis.

## Literature Review

### Crisis and Situational Crisis Communication Theory (SCCT)

Crises have been defined as “unpredictable events that can disrupt an organization’s operations and threaten to damage organizational reputations” [5]. The priority in any crisis is to protect stakeholders from harm; therefore, public safety concerns should precede any reputational concerns [6]. Stakeholders will attribute responsibility of the crisis to the company, which can be grouped into three categories: Victim (weak attributions of organizational responsibility such as natural disasters or rumors), accidental (low level of responsibility such as challenges or technical-errors), and preventable (high level of organizational responsibility such as human-error or organizational misdeed) [7]. As industries are the victims of the Coronavirus pandemic and could do nothing to avoid it, the response strategies for managing this crisis will be based on the victim category.

According to Coombs Situational Crisis Communication Theory (SCCT), for each of the attribution clusters mentioned, there are communication strategies to employ to protect company reputation in three ways: 1) shape attributions of the crisis, 2) change perceptions of the organization, and 3) reduce the negative effect generated by the crisis [8]. When the organization has been attributed with weak responsibility as seen with the Coronavirus, the organization should implement an ethical base response consisting of instructing information (i.e., explaining the crisis to the stakeholders) and adjusting information (i.e., helping stakeholders cope with the crisis). However, it could also include bolstering messages such as ingratiation (i.e., praising stakeholders for efforts they have made) and victimage (i.e., reminding stakeholders that the organization is a victim of this crisis) [9]. Unlike bolstering, which focuses on communicating past good works a company has done, enhancing includes telling stakeholders about the company’s current good works [10]. In the Coronavirus case, this could entail communicating the new safety measures put in place for both the employees and the customers. This paper examines how SCCT crisis responses were communicated online, particularly the enhancing strategy, by Swiss 4 and 5-star, independent hotels.

### Crisis Communication in the Hospitality Industry

Previous studies have examined crises in the hospitality industry that have significantly impacted specific properties or areas. From war and political instability to crime, terrorism, and health epidemics, safety concerns are a significant predictor of travel intentions [11]. Swiss hotels have circumvented major crises thus far. Their recent crises have included little or no snow during regular ski seasons, a hailstorm that destroyed some vineyards, and the financial burden of a strong Swiss franc. While researchers have recommended that hotels post messages regarding the crisis, irrespective of the crisis level, on their websites so that potential customers will read these messages when connecting to make their reservations [12], some hotels are reticent for fear of lowering the interest in their properties if their customers read messages about a crisis [9], particularly a health crisis like the Coronavirus. This study analyzes how many hotels effectively communicated these messages in the de-confinement stage of Swiss hospitality.

Previous studies have examined how and what companies communicate with their stakeholders in times of crisis. Specifically, in the hospitality industry, researchers have examined social media use through social media such as Twitter or Facebook and traditional media during crises. One study focused on the tourists’ risk perceptions associated with health amongst others to gauge how frequently tourists resort to social media to gather their information to find that international tourists with high-risk perceptions had a high likelihood of seeking information through social media channels during a crisis [13]. The perception, particularly of risk associated with crises, can be mediated by using social media to give information and address the human needs of conversation and compassion [14]. Nonetheless, international travelers are more vulnerable due to linguistic communication, culture, and varying levels of social media savvy, which may affect their choice to use social media to seek information in the event of a crisis [15]. As Switzerland is dependent on international travel for their tourism, particularly for hotel occupancy, this study will analyze the messages on both the official hotel websites and their Facebook pages and address the following two research questions:

RQ1: What are Swiss 4 and 5-star independent hoteliers communicating on their official websites regarding the Coronavirus?

RQ2: Are Swiss 4 and 5-star independent hoteliers using social media to communicate about the Coronavirus crisis?

## Methodology

**Fig. 1.**
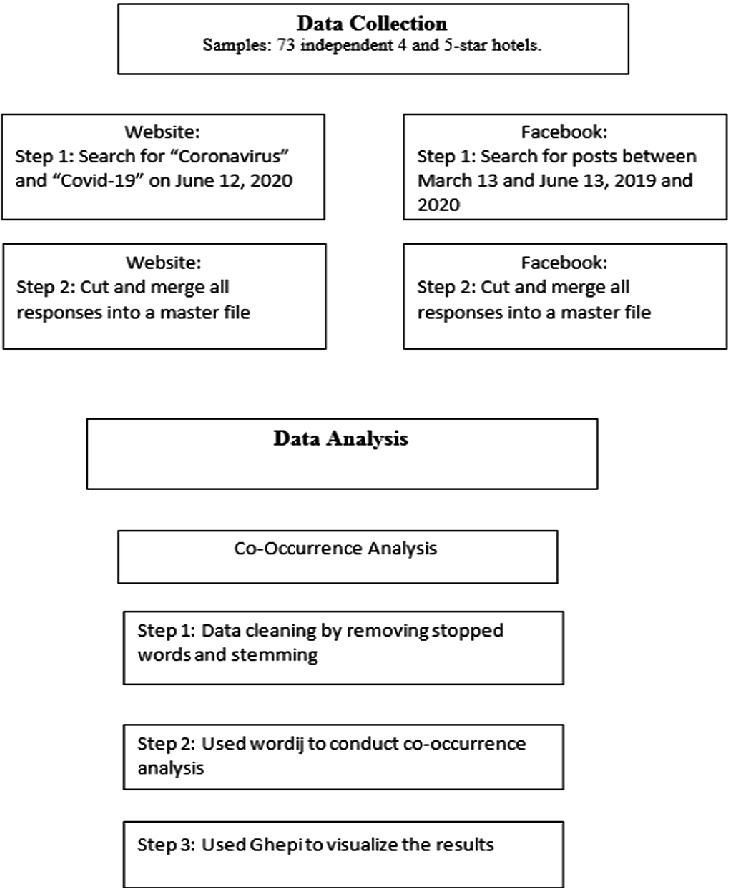
Research methodology.

A list of 4 and 5-star independent hotels in Switzerland was gathered to examine the messages posted on their official websites and Facebook pages. One criterion for inclusion in this study was total independence; thus, hotels that were part of chains or groups were not included. Of the 222 hotels on the original list, only 96 had Facebook pages. Of the 96, hotels that were part of a group (though not a chain) and hotels with no Facebook page were eliminated. Also, hotels that were not yet open were eliminated as the purpose was to analyze their reopening messages regarding the Coronavirus. The official company websites were accessed on June 12, 2020, when many hotels had officially opened. The Facebook comments were tracked from mid-crisis when many hotels were closed (March 13, 2020) to June 13, 2020, again, when many of the hotels reopened. Figure 1 shows the research methodology employed.

While Swiss hotels are located in various regions and are often set to the local language of the canton in which the hotel is situated (French, German, or Italian), most of these websites had a combination of the national languages, and all of them had an English language setting. This could be explained by the number of international tourists who peruse the official websites to book their accommodations. For this reason, the English setting was used on all of the official hotel websites, and the search for Coronavirus information was conducted. The homepage of each hotel website was scrutinized for direct messages that could be found. Some hotel websites offered a pop-up message with Covid-19 information directly on access.

Further, each website was searched using the keywords Coronavirus, and Covid-19 were entered into the search boxes when available. These two keywords were chosen to establish timely messages regarding the pandemic. While health and safety measures could have been used, hotels already have best practices in place. The purpose of this study was to identify the ‘new’ messages written precisely in response to the Coronavirus. For the Facebook pages, again, the messages posted in English were chosen for this research project to remain consistent with the international clientele who seeks information via the hotels’ websites.

## Results

### Hotel Official Websites

**Fig. 2.**
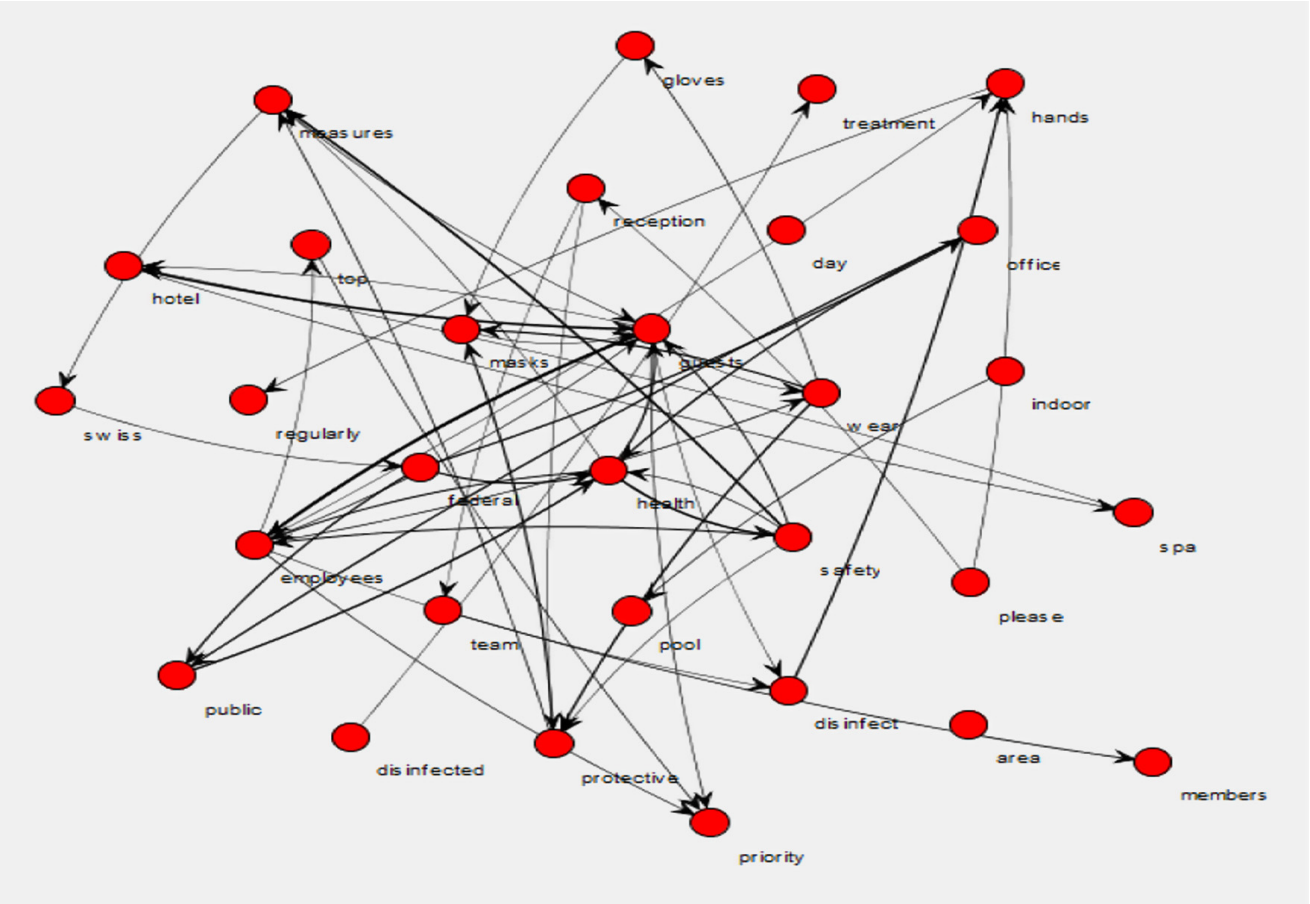
Wordji image of the messages on the official hotel websites.

The official hotel websites offered a varied amount of information regarding the Coronavirus. Figure 2 shows the relationships between the words in the messages found on the hotel websites. Twenty-nine of the hotels had a direct message, sometimes a pop-up, which appeared on their homepage regarding Coronavirus or Covid-19. Word pairs such as ‘federal office,’ ‘federal public,’ ‘federal health,’ ‘office health,’ and ‘office public’ appeared 12 times in the official hotel website messages. ‘Disinfect hands’ and ‘wear protective’ appeared 13 times before ‘wear masks’ and ‘wear mask’ (at 12 and 10 times, respectively). The highest number of word pairs was attributed to ‘guests employees’ (23 times), followed by ‘hygiene measures’ (18 times), ‘hotel guests’ (17 times), ‘safety measures’ (16 times), ‘health safety’ (15 times), and Public health’ and ‘public areas’ (14 times). The number of unique pairs was 363, with an average pair frequency of 4.440771 and pair entropy (or level of uncertainty or randomness) of 5.765317.

For the frequency of individual words, the most common word in the messages were ‘guests’ (126 times). The word ‘hotel’ (81 times) was followed by ‘employees’ (58 times), ‘safety’ (53 times), ‘measures’ (52 times), ‘hygiene’ (40 times), and ‘distance’ (34 times). The majority of the individual words were tabulated less than ten times. Out of 440 unique words, 342 (77%) were used less than ten times in all of the messages combined. The average word frequency was 8.1, and the entropy was 5.690377.

### Hotel Facebook Comments and Hashtags

**Fig. 3.**
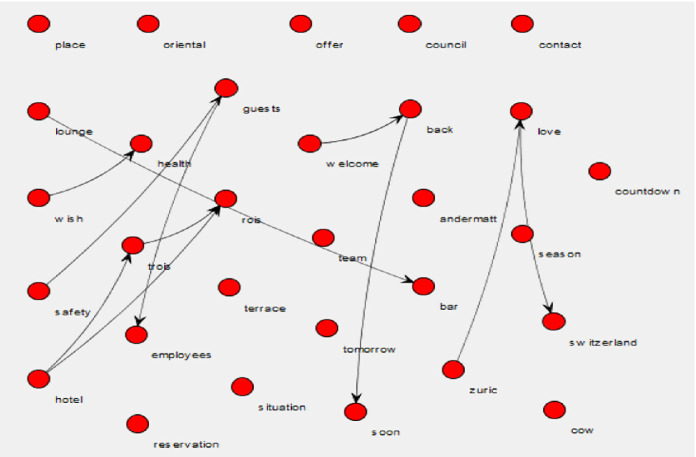
Wordji image of the words used in the messages posted on Facebook.

The messages on Facebook were also analyzed both for the individual words used and the word pairings. Figure 3 shows the relationship between unique words (71) found in the Facebook posts. Only three word pairs appeared ten or more times in the analysis: ‘forward welcoming’ (15 times), ‘forward back’ (10 times), and ‘dear guests’ (10 times). As seen in Fig. 3, trio relationships can be seen through words such as ‘safe,’ ‘health,’ and ‘employees,’ ‘welcome,’ ‘back,’ and ‘soon,’ and ‘love,’ ‘Switzerland,’ and ‘Zurich.’ The only words that specifically refer to the Coronavirus pandemic were ‘health’ and ‘safety.’ The average pair frequency was 4.450704, and the pair entropy was 4.174661.

**Fig. 4.**
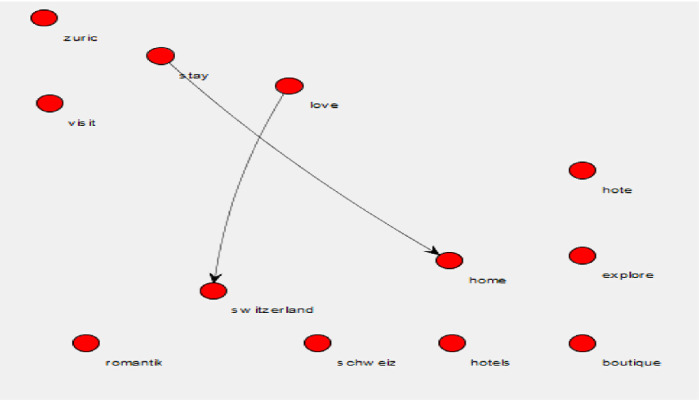
Wordji image of the words used in the hashtags.

Figure 4 shows the results from the hashtags associated with the Facebook comments.

As seen in Fig. 4, the relationships between the words used in the hashtags were also examined. Relationships were established between ‘Switzerland’ and ‘love’ and ‘stay’ and ‘home.’ Only ‘stay’ and ‘home’ could be linked to the current Coronavirus pandemic. Further, just ten unique word pairs appeared less than five times in the hashtags (average pair frequency of 3.2). The pair entropy was calculated as 2.295203. The individual words found in the hashtags resulted in 20 unique words for an average word frequency of 4.15 and entropy of 2.91938.

## Discussion

For the official hotel websites, only 29 out of the 73(39%) 4 and 5-star independent hotels examined in this study published a message regarding Coronavirus measures or corrective actions on their sites. As mentioned in the literature review, this could be explained by the fact that this was not a unique crisis affecting an individual property or region; instead, this crisis affected all hotels and regions equally. There was minimal responsibility attribution allocated to the hotels; thus, their response could be justifiably minimal. However, in this study, the vast majority of the hoteliers seemed to employ the *ignore* strategy of crisis communication, i.e., if we do not talk about it, it will go away on its own. Hoteliers may not have felt the need to remind guests of the pandemic at a time when they were trying to reserve a holiday to escape the last few months of worry. Out of sight, out of mind could have been the strategy employed by these hotels. Further, there was little, if any, use of the *victimage* strategy, i.e., reminding customers that the hotels are victims of this crisis. This could be explained by the fact that the customers are victims in this pandemic; thus, one victim telling another victim that they are a victim could be counter-productive.

RQ1: What are Swiss 4 and 5-star independent hoteliers communicating on their official websites regarding the Coronavirus?

Compared to social media communication, some hoteliers did communicate information regarding the Covid-19 pandemic. As seen in Fig. 2, the hoteliers who did publish messages that focused on hygiene, safety, health, and protective measures implemented part of Coombs’ base response of SCCT, i.e., providing instructing information or giving information about the situation to the stakeholders. The hotels also employed the enhancing strategy or telling guests the ‘good’ actions they are taking, such as disinfecting areas and wearing masks. While the instructing information aligns with the base response of SCCT, the adjusting information, i.e., that of the caring response, appeared to be missing from these messages. This is not to say that it never appeared in any message; instead, it did not appear often enough to be significantly recognized. At times, the hoteliers referred to external third parties, such as the Swiss government, to justify the measures they were taking. Nonetheless, no hotel over-emphasized the measures taken by the hotel. This could be explained in two ways: 1) hoteliers did not want to increase the risk perception of travel for potential tourists, or 2) hoteliers are reticent to discuss negative situations when promoting the positive side of staying in their hotels.

The messages communicated on social media (Facebook) pages contained much less information regarding the Coronavirus. The focus on the Facebook messages was on welcoming back the clientele after a long period of closure and included words of the services they offer, such as ‘bar’, ‘lounge’, and ‘terrace’. The hoteliers posted messages that were upbeat and positive as if trying to convince the clientele that the opportunity to return to their properties had finally arrived. These messages did not inform the guests that the experience could potentially be different from previous stays; instead, the messages focused on getting the clients back. This could be interpreted as an application of the reminder strategy, i.e., reminding the clientele of the great experience they had the last time they stayed in the hotel. The hashtags echoed this same spirit. Even the hashtag ‘stay home’ could be interpreted as staying in the home country, this case, Switzerland, i.e., targeting local customers who will choose domestic destinations this year instead of traveling abroad.

RQ2: Are Swiss 4 and 5-star independent hoteliers using social media to communicate about the Coronavirus crisis?

The response to this question confirms previous results on how hotels effectively or ineffectively use social media at any time, crisis, or not. To summarize, there is a lack of interactive dialogue between Swiss hoteliers and their guests via social media. The results from this study demonstrate that hoteliers were not using social media, at least not Facebook, to communicate their actions in regards to the Coronavirus pandemic. Although the literature suggested that international travelers are the most likely to use social media to gather information in times of crisis and most hotel guests in Switzerland are international, this study showed that hoteliers in Switzerland did not provide this information through the social media channels. Hoteliers may have missed an opportunity to show compassion or empathy that is so important during any crisis, regardless of attribution level.

## Conclusion/Implications

Coombs’ SCCT outlines the importance of preparing strategic messages that align with the crisis a company faces by establishing the level of attribution, the past crises a company has faced (i.e., a history of crises), and the reputation a particular company has in dealing with crises. Based on the SCCT theory, crisis communication teams can brainstorm potential crises for their industry and proactively prepare crisis communication templates for each type of potential crisis. Before Covid-19, the hospitality industry has endured other global health crises such as severe acute respiratory syndrome (SARS) and the Middle East Respiratory Syndrome (MERS), as well as terrorist attacks, hurricanes, and tsunamis. However, these crises affected one property, one region, one industry, or one country. A pandemic such as the Coronavirus that affected the entire hospitality industry worldwide has yet to be seen in this century.

Unlike previous crises, this pandemic has made it impossible to claim the ‘victim’ status. As seen in this study, hotels cannot play the victim card because their competitors are equally victims. While taking precautions and communicating them to the clients, some hotels may be protecting themselves against the worst-case scenario, i.e., an outbreak in their hotel that affects their clients and employees. In the case of an outbreak, the ‘silent’ hotels may be attributed responsibility as the ‘victim’ crisis would be perceived as a ‘preventable’ one. Hoteliers need to make strategic decisions on how many corrective actions are put in place and how they will communicate these actions to the stakeholders. Without such, hotels are putting themselves and their reputations at risk for the future.

From March to June, the number of new cases of Covid-19 dropped to approximately a dozen; the number of hospital cases and deaths have declined as well. Unfortunately, by mid-June, the number of new cases began to rise again. On August 12, the announcement that events with more than 1000 people would again be permitted on October 1, a new spike in cases has reversed this decision. On October 18, the Swiss government announced a new set of measures beyond social distancing and hygiene rules, such as mandatory mask-wearing in all public places, including train stations, airports, shops, and restaurants [16].

Since the completion of the initial phase of this study on domestic tourism, Swiss tourism has survived the summer season. In an article published by swissinfo.ch on October 21, 2020, the authors summarized what has happened since June. According to the Federal Statistical Office, domestic guests helped fill the void of international guests for Swiss hotels, which fell by 60.3%. Compared to August 2019, Swiss hotels in 2020 welcomed more domestic guests (approximately 1/6 more than the previous year). Nonetheless, by mid-August, the Swiss hotel industry slowed down, with hotel occupancy declining by 28.1% compared to 2019. Overall, the Swiss hotel industry witnessed a 40.9% decline in overnight stays in the first eight months of 2020, mainly by international travelers (down by 61.7%) [16]. At the moment, with the new spikes in and the second wave of Covid-19 cases, the future looks bleak for Swiss tourism and, particularly, Swiss hotels. Unless Swiss hoteliers can convince their customers to return to their hotels, Mr. Zullig’s predictions, i.e., the closing of 5% of Swiss hotels, may become a reality.

## Limitations/Future Studies

This study had several limitations. Firstly, the hotels were limited to 4 and 5-star properties that are independent and had Facebook pages. Future studies could evaluate all categories of hotels in Switzerland to gauge how other categories communicated their pandemic measures to their customers. Secondly, the language used for analysis was English. Although justified by the international clientele that most frequents these establishments, further studies could analyze the other languages and their messages. Thirdly, the study was limited to Swiss hotels. Comparative studies against other countries could be preferable to identify best practices in communicating about a crisis on an industry level. Fourthly, the analysis of this study’s message was one-sided, deriving only from the hotels’ official websites and Facebook pages. A further study should investigate the dialogue between the hotels and the stakeholders. Other social media channels such as Twitter or Instagram could be analyzed as well. Finally, this study did not investigate messages they may have been communicated within the hotels upon arrival. Hoteliers may have had posters or brochures at the reception, in the rooms, or in the public spaces outlining the hygiene and safety measures guests were expected to follow.
